# Setting the table for meat consumers: an international Delphi study on in vitro meat

**DOI:** 10.1038/s41538-019-0041-0

**Published:** 2019-06-04

**Authors:** Victor Tiberius, Jenny Borning, Sabrina Seeler

**Affiliations:** 1 University of Potsdam, August-Bebel-Straße 89, 14482 Potsdam, Germany; 2 Auckland University of Technology, 55 Wellesley Street East, Auckland City, New Zealand

**Keywords:** Economics, Protein delivery, Sustainability

## Abstract

The growing global demand for meat is being thwarted by shrinking agricultural areas, and opposes efforts to mitigate methane emissions and to improve public health. Cultured meat could contribute to solve these problems, but will such meat be marketable, competitive, and accepted? Using the Delphi method, this study explored the potential development of cultured meat by 2027. Despite the acknowledged urgency to develop sustainable meat alternatives, participants doubt that challenges regarding mass production, production costs, and consumer acceptance will be overcome by 2027. Considering the noticeable impacts of global warming, further research and development as well as a change in consumer perceptions is inevitable.

## Introduction

Global meat consumption has been increasing in recent decades, though with regional differences: while average per capita consumption has been stagnating or even decreasing in many high-income countries, it has risen drastically in middle-income countries.^1^ This positive correlation between disposable income and consumption of meat and dairy products—termed Bennett’s law—has been confirmed in previous research.^2^ In addition, meat choices are changing and the consumption of processed meat and meat alternatives is rising.^3^

With the global population growth and a rising middle-class, global meat demand is expected to accelerate even faster.^4^ If this growing demand is to be met, the area of agricultural land will have to increase as well. Due to climate change, degradation, erosion, and pollution, the land area available for agricultural use is actually shrinking, with a loss of nearly one-third of the world’s arable land since the 1970s.^5^ With more than half of agricultural emissions arising from enteric fermentation, an increase in the livestock population, particularly ruminant animals such as cattle and sheep, would substantially raise the emissions of CO_2_ and methane.^6^ Ecotrophologists are also concerned with negative impacts on consumer health, especially of increased red meat consumption.^4^ These prospects call for the urgent search for alternatives to conventional meat production.

Plant-based proteins (e.g., proteins based on soy or lupines) are mainly consumed by vegetarians and vegans as smaller market segments but are insufficiently accepted by the general public.^7^ Insects as another source of protein are increasingly used in some African, Asian and Latin American countries, while Western markets are more hesitant with this kind of nutrition. Although the majority of consumers remain omnivores, they are generally reluctant to adopt alternative diets.^8^ Meat consumption seems to be deeply imprinted in consumers’ value systems evoking strong emotional connections and providing a satisfying experience for all the senses.

With the aim of satisfying the demand for meat, and at the same time minimizing its negative impacts on the environment and health, researchers have explored laboratory-grown meat as an alternative, so-called cultured or in vitro meat.^9^ A form of cellular agriculture is applied in which animal cells are cultured with the help of tissue engineering or 3D printing technologies.^10^ The Dutch scholar Mark Post demonstrated the first successful proof-of-concept in 2013. Over a period of two years and with an investment of US$300,000, Post cultured beef from thin strips of fresh muscle to produce an 85 g cultured beef burger.^11^

The production of cultured meat is still in its infancy and further research and development is required to realize a competitive alternative that is generally accepted.

To date, a broad market launch has not taken place and cultured meat is not yet commercially available. Thus, the actual acceptance level of cultured meat is unclear and it remains unanswered whether cultured meat will become a niche or mainstream product. While cultured meat experts propagate the multiple advantages of the product unanimously, consumers have a more differentiated view.^12,13^ Several consumer surveys have investigated attitudes towards cultured meat with equivocal results on the personal and societal level. While most US consumers would try cultured meat once, they cannot imagine replacing farmed meat regularly.^14^ European consumers who gained more technical knowledge about cultured meat production even reacted with disgust.^15^ Consumers expect a high price and a limited taste.^14,16^ In addition, the synthetic nature of cultured meat contradicts the global trend for natural food.^13–15,17^

When taking a societal perspective, consumers have a more positive attitude as they understand the negative ecological, ethical, health, and food safety concerns related to factory farming.^15,18^

Acceptance of cultured meat strongly depends on demographics and individual values. For example, men have a stronger tendency to cultured meat than women.^14^ Educated consumers do not expect cultured meat to be a sustainable solution for the problems with factory farming.^19^ Muslims can accept cultured meat if it is halal.^20^

## Results and discussion

### Descriptive Statistics

The respondents agreed to projections 2, 5, 6, 7b, 15, 17, 19, 20, 23, and 24 and partly agreed to projections 9 and 21, while projections 1, 3, 8, 10–14, 18, and 22 were rejected. Due to the high scattering of the responses and several commentaries, the expert opinions regarding projection 4, 7a, 16, 21, and 25–31 do not provide a unified picture (Table 1).

Comparing the results of the first and second round, the scattering for ten projections—1, 3, 10–14, 17, 18, 23—decreased. The highest reduction and, therefore, consensus can be found for the environment-related projections. Critics argue that a decrease of IQR represents a majority impact and the tendency to the group median rather than an approximation to the most-probable future development.^21–23^ For projections 4, 6, 8, 25, 27, and 30, the IQR increased unusually indicating a high level of uncertainty among the respondents. Only a few slight shifts of the median occurred. Since projection 7b was added for the second round, a comparison with the first round is omitted.

**Table 1:** Descriptive statistics

Projection		1st Round (*N* = 37)				2nd Round (*N* = 30)				Difference			
		*x*_0.25_	*x*_0.5_	*x*_0.75_	IQR	*x*_0.25_	*x*_0.5_	*x*_0.75_	IQR	*x*_0.25_	*x*_0.5_	*x*_0.75_	IQR
*Cluster 1: Technology*													
1	Lower production costs	2	2	4	2	2	2	3	1	0	0	−1	−1
2	Mass production: Bioreactors	3	4	4	1	3	4	4	1	0	0	0	0
3	Mass production: 3D print technology	2	2	4	2	2	2	3	1	0	0	−1	−1
4	Mass production: different methods	3	3	4	1	2	3	4	2	−1	0	0	+1
5	Production without fetal calf serum	3	4	4	1	3	4	4	1	0	0	0	0
6	Enrichments with vitamins etc.	4	4	5	1	3	4	5	2	−1	0	0	+1
7a	Government subsidies	2	3	4	2	2	4	4	2	0	+1	0	0
7b	Cooperations	–	–	–	–	3	4	5	2	–	–	–	–
8	Production at home possible	1	2	2	1	1	2	3	2	0	0	+1	+1
9	Structured, cultured meat possible	2	2	4	2	2	3	4	2	0	+1	0	0
*Cluster 2: Environment*													
10	Reduction of greenhouse gas emissions	0	5	20	20	0	1.5	10	10	0	−3.5	−10	−10
11	Reduction of agricultural spaces	1	5	20	19	0	5	10	10	−1	0	−10	−9
12	Reduction of livestock	0	5	20	20	0	3	10	10	0	−2	−10	−10
13	Increase of energy consumption	0	0	5	5	0	0	2	2	0	0	−3	−3
14	Reduction of water consumption	0	5	20	20	0	1	10	10	0	−4	−10	−10
15	Environmental advantages/ disadvantages proved	3	4	4	1	3	4	4	1	0	0	0	0
16	Other risk with negative effects	2	3	4	2	2	2.5	4	2	0	−0.5	0	0
*Cluster 3: Market and competition*													
17	Niche product	3	4	4	1	4	4	4	0	1	0	0	−1
18	Higher market share than other meat substitutes	1	2	3	2	2	2	3	1	1	0	0	−1
19	Intensified competition among producers	3	4	4	1	3	4	4	1	0	0	0	0
20	Sales to meat processing industry/manufacturer	3	4	4	1	3	4	4	1	0	0	0	0
21	Production by conventional meat producers	2	3	4	2	2	3	4	2	0	0	0	0
*Cluster 4: Consumers*													
22	Price advantage over conventional meat	2	2	3	1	2	2	3	1	0	0	0	0
23	Price-independent factors	2	4	4	2	4	4	4	0	2	0	0	−2
24	Acceptance due to safety standards	3	4	4	1	3	4	4	1	0	0	0	0
25	Perception as healthy product	3	4	4	1	2	4	4	2	−1	0	0	+1
26	Acceptance due to transparency	3	4	4	1	3	3.5	4	1	0	−0.5	0	0
27	Acceptance due to animal welfare	3	4	4	1	2	4	4	2	−1	0	0	+1
28	Acceptance due to environmental factors	2	4	4	2	2	4	4	2	0	0	0	0
29	Equivalent in appearance	2	3	4	2	2	4	4	2	0	+1	0	0
30	Equivalent in taste	3	3	4	1	2	4	4	2	−1	+1	0	+1
31	Equivalent in meat texture & structure	2	3	4	2	2	3	4	2	0	0	0	0
*N*—sample size	x_0.25_—lower quartile	*x*_0.5_—median				*x*_0.75_—upper quartile				IQR—interquartile range			

### Scenario

#### Advancing innovative technologies

Although production costs have fallen since the first beef patty was introduced in 2013, experts are not convinced that reduction in costs will be sufficient to make cultured meat price-competitive with conventional meat by 2027. Experts are also not convinced that 3D printing technologies will develop fast enough to facilitate mass production of cultured meat, meaning that its accessibility will remain limited in the next years. Whether alternative mass production methods, such as bioreactors, will be developed that allow efficient cultivation of meat that is more marketable and competitive is disputed. Experts are optimistic that improved production processes will create cultured meat free from animal by-products that might be more nutritious and healthier than conventional meat, though such an achievement is expected to depend strongly on public subsidies and stakeholder cooperation.

#### Increasing market size

Similar to the development of other plant-based alternatives that currently appeal to a rather small market segment, experts see cultured meat as a niche, not as a mass product. As meat substitute alternatives are already established whose market share is growing, it is considered unlikely that cultured meat will capture a higher market share than these by 2027. This could prove to be critical considering that huge investments have been made and will be needed in the future to realize the aspired environmental and health improvements ascribed to cultured meat. The sustained progress in research and comparably rapid development has recently attracted new investors. With the transition from research project to business stage, several start-ups have been founded aiming to become pioneers in the market. This will lead to intensified competition and correspondingly more media attention in the upcoming decade. In addition, a spillover effect of raising consumer awareness and stimulating demand could be expected.

#### Hardly any effect on the environment

Due to the low market share, only small positive effects on the environment will be seen. Greenhouse gas emissions, the size of agricultural spaces, the quantity of livestock, and water consumption will not significantly decrease. However, the experts do not expect an increase in energy consumption due to cultured meat production either. Researchers will be able to evaluate the effects on the environment better in the future.

#### Influencing consumer acceptance

As cultured meat is seen as unlikely to possess a price advantage, competitiveness can only be realized through non-price-related determinants, such as health features. Due to the product’s novelty and consumer inexperience with cultured meat, respondents showed uncertainty whether factors related to consumer health, transparency, animal welfare, or environmental conservation would be central to encourage acceptance and the willingness to purchase. High safety and health standards as well as transparent production processes are considered as an absolute requirement for cultured meat to be approved by consumers. Without improved control systems, the overall notion of developing a competitive substitute for conventional meat will not be accomplished. Closely related to the necessity of transparency in production processes is the pressing need to create a mass-market product that resembles conventional meat regarding cooking and sensory experiences.

If one were to plot the story of global warming as a dramatic play, the year 2018 would portray the second stage in Freytag’s five-stage dramatic structure: the “rising action”.^24^ The Northern Hemisphere has experienced a summer as never before—temperature extremes, record heat waves, wildfires, droughts, floods, crop failures. The impacts of global warming through greenhouse gas emission have been served on a silver platter. This seems to be a foretaste of what is to come, as the climax of the dramatic play has not been reached. If global meat consumption and production remain unchanged in the near future, the “falling action” might not resolve in the release of tension desired by today’s political leaders, scientists, and the general population.

The production of cultured meat should mitigate methane emissions. To achieve the aim of ‘a meat alternative for all’ that positively contributes to environmental conservation and health issues, cultured meat can only be a successful substitute if technological advancements are achieved. Results from this study demonstrate that experts are hesitant of projecting any substantial positive change in the next decade. The realization of mass production under the same conditions as small-scale laboratory tests is required and the cooperation and coordinated efforts of stakeholders, governmental support, and investors are crucial. It is seen as being very unlikely that production costs will decrease significantly in the near future and it is deemed doubtful whether technological advancements will result in the desired improvements by 2027. Most critical to the vital change in meat consumption, however, is the human factor and the uncertainty whether burgeoning demand will bring acceptance of cultured meat as an alternative to traditional meat.

With experts being skeptical of breakthrough improvements by 2027, and with the outlook being gloomy, a resounding success in sustainable meat production does not seem reachable, and a change in consumer perceptions and acceptance appears doubtful. However, the consequences of projections are not inevitable; they can be changed. As in any dramatic play, it will be the protagonists’ tasks to facilitate interventions and policies that will result in the desired outcome. The specific manner of communication is key in fostering consumer acceptance. Bekker et al. showed in their experiments that framing and priming could change customers’ explicit attitudes toward cultured meat significantly—even an established indirect cognitive association of cultured meat with solar panels could ameliorate the attitude toward cultured meat.^25^ Also positive imagery and narratives can foster a positive attitude.^26^

In addition, governments have to meet today’s regulatory challenges^27^ and could act on both the production and demand sides. Funding could facilitate research into cheaper mass production. As information on cultured meat and its benefits produce favorable expectations about the concept,^16^ public information could be more widely broadcast.^28^ Distorted images of obstacles in the way of cultured meat have to be removed from the recipients’ minds.^13,29^ Protagonists need to be reminded that change is slow, and might be even slower when it comes to products that are loaded with memories and personal attachment—replacing turkey with ham on Thanksgiving or at Christmas will require time, but that time will be well invested in the future.

## Methodology

### The Delphi method

Due to the fact that cultured meat has not yet been introduced to the market, it is necessary to use forecasting methods to build a reliable foundation for exploring the future of cultured meat.

To generate the aspired future scenario of the potential development of cultured meat by 2027, the Delphi study was conducted online. The Delphi method addresses the questioning of experts in the field of inquiry in at least two rounds using a standardized questionnaire and giving structured feedback about the results from the first round in order to enhance consensus among the respondents.^21,30–33^

The Delphi method is suitable for forecasting man-made future states.^33^ In contrast to causal-deterministic natural development processes, like weather, societal futures are based on human intentions, social interactions, and coincidence. Therefore, societal forecasts can be derived from a group of experts expressing their subjective, knowledge-based, and experience-based opinions, unlike laypersons’ opinions.

In contrast to scenario analyses,^34,35^ whose intent is to generate multiple future scenarios, the Delphi method aims at the current most-probable scenario which is generated by merging multiple expert statements.

After the first questioning, the interim results of the first round are revealed to the respondents during round two. Some scholars criticize that the scientific requirement of independent judgments is violated by this disclosure.^36,37^ However, the aim of the Delphi study is the convergence of expressed opinions and the narrowing of the statistical spread to receive an unequivocal scenario as a consensus. Conclusions for the resulting scenario can be drawn from consensus or from a dissent.

The Delphi study was conducted in accordance to the voluntary “Research guidelines for the Delphi survey technique”.^38^ Since the survey did not include any medical or clinical experimentation or ethically relevant human research, the ethics commission of the University of Potsdam was not competent for this kind of research and an approval was neither needed nor possible.

### Selection of panelists

The selection of appropriate respondents for the Delphi study is often confronted with the question of how an expert can be characterized, and how his or her expertize can be measured.^39^ For this study, 82 potential respondents who had published professionally on cultured meat were identified. The experts work at universities or other research institutions, start-up companies researching or promoting cultured meat, foundations, and state institutions from the United States of America, The Netherlands, Germany, and several other countries. Of this sample, 39 participated in the first round, 30 in the second round. Although the sample size appears relatively limited, Delphi studies are usually small-scale and considering the infancy of the field, the sample size is satisfactory for this research project.

The participants were informed that their provided information would be anonymized and that the results of the survey would be published. Written consent was obtained from the participants.

### Data collection

The questionnaire contained 31 projections regarding possible technological developments, their impacts on the consumer and the market, and competitiveness derived from a literature review and exploratory interviews.

To minimize the risk of misinterpretations of the statements presented to the experts, a pretest was conducted. The respondents were asked to express their consent or dissent with most projections on a five-point Likert scale (“do not agree at all”, “rather do not agree”, “partly agree”, “rather agree”, and “agree completely”). For projections 10–14, the respondents were asked to provide estimates in percentage. Projection 7b was added after the first round due to suggestions from respondents (Table 2).

The study was conducted anonymously, since anonymity is regarded a central feature of Delphi studies.^33^ Critics argue that anonymity is accompanied by the respondents’ irresponsibility with the results.^22,37^ This is especially a problem when self-fulfilling prophecies are expected. However, in this study it can be expected that scientific progress has a higher impact on the future development than the expression of the experts’ views.

**Table 2:** Delphi projections

No	Projection: By the year 2027, …	Response type
*Cluster 1: Technology*		
1	production costs of cultured meat will be significantly lower compared to conventional meat.	Likert
2	mass production of cultured meat will be possible due to further development of bioreactors.	Likert
3	mass production of cultured meat will be possible due to 3D printing.	Likert
4	mass production of cultured meat will be possible due to another method which is not utilized today.	Likert
5	there will be at least one mature alternative to produce cultured meat in larger quantities without fetal bovine serum.	Likert
6	cultured meat can be enriched with more vitamins, minerals, and poly-unsaturated fatty acids than conventional meat.	Likert
7a	it will have become possible with the help of government subsidies to refine the production process for cultured meat.	Likert
7b	the cooperation between research teams, market participants (like start-ups or investors), and government will have improved to refine the production process for cultured meat.	Likert
8	consumer will be able to produce cultured meat at home.	Likert
9	it will be possible to produce highly structured cultured meat (e.g., steak meat).	Likert
*Cluster 2: Environment*		
10	the greenhouse gas emissions caused by agriculture can be reduced worldwide by about X percent.	Percentage
11	the agricultural land used for livestock breeding and feed crop can be reduced worldwide by about X percent.	Percentage
12	the number of farm animals can be reduced worldwide by about X percent.	Percentage
13	the energy requirement or meat production will increase by about X percent.	Percentage
14	the required quantity of water for meat production will be reduced by about X percent.	Percentage
15	environmental advantages and disadvantages of cultured meat will have been sufficiently investigated and proven.	Likert
16	other risks will have been identified in the production process of cultured meat which will have a negative impact on the environment.	Likert
*Cluster 3: Market and competition*		
17	cultured meat will be a niche product with a small market share (less than 10 percent).	Likert
18	cultured meat will have a larger market share than other meat substitutes (like products made of soy, lupines, or insects).	Likert
19	There will be an intense worldwide competition between several companies producing cultured meat.	Likert
20	companies will exist that are specialized in the production of cultured meat to sell it to meat processing companies (e.g., sausage manufacturers).	Likert
21	conventional meat producers will also produce cultured meat.	Likert
*Cluster 4: Consumers*		
22	the demand for cultured meat will be positively influenced because it can be offered at a lower price than conventional meat.	Likert
23	the demand for cultured meat will be positively influenced by offering more attractive non-price aspects in cultured meat to consumers.	Likert
24	consumer acceptance will increase due to high security standards and relevant certifications in the production process of cultured meat.	Likert
25	consumers will appreciate cultured meat as a healthy product (due to added nutrients).	Likert
26	a special product label will enhance transparency for consumers and thus the acceptance of cultured meat will increase.	Likert
27	cultured meat will be better socially accepted than conventional meat due to animal welfare regards.	Likert
28	cultured meat will be better socially accepted than conventional meat due to the reduction of environmental impacts.	Likert
29	cultured meat will be equivalent to conventional meat in appearance.	Likert
30	cultured meat will be equivalent to conventional meat in taste.	Likert
31	cultured meat will be equivalent to conventional meat in meat texture.	Likert

### Reporting summary

Further information on research design is available in the Nature Research Reporting Summary linked to this article.

## Supplementary information

Reporting Summary

## Data Availability

The authors confirm that all relevant data are included in the paper. Further data available on request from the authors.

## Supplementary information

**Supplementary information** accompanies the paper on the *npj Science of Food* website (10.1038/s41538-019-0041-0).
